# The Contribution of Extracellular Vesicles From Senescent Endothelial and Vascular Smooth Muscle Cells to Vascular Calcification

**DOI:** 10.3389/fcvm.2022.854726

**Published:** 2022-04-15

**Authors:** Cristina Mas-Bargues, Consuelo Borrás, Matilde Alique

**Affiliations:** ^1^Grupo de Investigación Freshage, Departamento de Fisiología, Facultad de Medicina, Universidad de Valencia, Valencia, Spain; ^2^Instituto Sanitario de Investigación INCLIVA, Valencia, Spain; ^3^Centro de Investigación Biomédica en Red Fragilidad y Envejecimiento Saludable, Instituto de Salud Carlos III (CIBERFES, ISCIII), Madrid, Spain; ^4^Departamento de Biología de Sistemas, Universidad de Alcalá, Madrid, Spain; ^5^Instituto Ramón y Cajal de Investigación Sanitaria (IRYCIS), Madrid, Spain

**Keywords:** aging, aging-related diseases, extracellular vesicles, inflammation, medial arterial calcification, senescence, smooth vessel cells, vascular calcification

## Abstract

Vascular calcification is an irreversible pathological process associated with a loss of vascular wall function. This process occurs as a result of aging and age-related diseases, such as cardiovascular and chronic kidney diseases, and leads to comorbidities. During these age-related diseases, the endothelium accumulates senescent cells, which stimulate calcification in vascular smooth muscle cells. Currently, vascular calcification is a silent pathology, and there are no early diagnostic tools. Therefore, by the time vascular calcification is diagnosed, it is usually untreatable. Some mediators, such as oxidative stress, inflammation, and extracellular vesicles, are inducers and promoters of vascular calcification. They play a crucial role during vascular generation and the progression of vascular calcification. Extracellular vesicles, mainly derived from injured endothelial cells that have acquired a senescent phenotype, contribute to calcification in a manner mostly dependent on two factors: (1) the number of extracellular vesicles released, and (2) their cargo. In this review, we present state-of-the-art knowledge on the composition and functions of extracellular vesicles involved in the generation and progression of vascular calcification.

## Introduction

Vascular calcification (VC) is a well-established multifactorial disorder characterized by calcium deposits along the vascular wall (1, 2). However, the calcification of vascular structures is unclear. Internal structures such as smooth muscle cells of the vascular wall undergo calcification that confers them osteoblast-like characteristics (3, 4). This prevents valvular or vascular adaptability and favors the development of pathologies. In general, there are three types of VC: medial arterial calcification, intimal calcification, and infantile calcification (2). This review mainly focuses on medial arterial calcification, which is often related to old age, diabetes mellitus, and cardiovascular diseases (CVD) associated with chronic kidney diseases (CKD) (5). In contrast, intimal calcification is only observed in patients with CVD such as atherosclerosis and hypertension (6). Infantile calcification refers to general arterial calcification in infants that is characterized mainly by medial calcification (7). Moreover, hydroxyapatite crystals could appear in cardiac valves (heart valve calcification) (6). Medial arterial calcification consists of hydroxyapatite crystals, which are calcium and phosphate mineral deposits found in bones (8–11). This type of calcification is a characteristic of CKD patients with CVD (6, 12–15). The pathology of medial arterial calcification is multifactorial (12). It is a gradual process resulting from disruptions in calcium, phosphate, and vitamin D homeostasis (8, 16). The pathophysiological process of vascular wall calcification involves some features of bone morphogenesis. Different cellular and molecular mediators are also involved such as pro-inflammatory molecules and molecules contributing to oxidative stress (8). Recently, extracellular vesicles (EVs) have been shown to play a role (17–21).

Chronic inflammatory diseases, including CKD, are considered synergistic pathologies due to the high risk of comorbidities, including CVD (12, 22–24). Furthermore, aging precipitates the appearance of age-related pathologies such as CKD and/or CVD, and vice versa; thus, these chronic inflammatory diseases trigger premature aging (19, 23). In general, the role of calcification in aging and age-related pathologies remains unclear. Calcification appears early and progresses rapidly, constituting a severe complication of kidney disease. Thus, VC is considered the primary cause of cardiovascular morbidity and mortality in patients with CKD. Due to its complex development mechanism, there are few tools for CKD treatment (22, 25). In this context, according to what we know so far, it would be essential to study the role of new mediators such as EVs, which are involved in aging, age-related diseases, and vascular calcification (18, 26, 27).

Vascular calcification generates complications in numerous cardiovascular pathologies. Coronary artery calcification is highly prevalent in CKD and coronary atherosclerotic plaques (5, 6, 28). Such calcification is classified as an intimal and medial calcification depending on the specific risk factor and is associated with major cardiovascular events (28). In the case of patients with acute myocardial infarction, they develop coronary calcification, which is associated with a high mortality rate (29). Furthermore, calcification is the most prevalent valvular disease in western societies, and calcific aortic stenosis is associated with a prevalence of 2% older (from 65 years) (30). Vascular calcification is also involved in aortic diseases, especially the ascending aorta called the porcelain aorta (31, 32).

Evidence suggests that chronic inflammation is a central factor in calcification. In the vasculature, chronic inflammation triggers atherosclerotic calcification (33). Subsequently, VC generates abnormalities in arteries, such as changes in blood flow due to the decreased wall elasticity and increased arterial stiffness, which decrease end-organ perfusion and cause injury (1, 8, 12). Consequently, VC may cause vascular alterations, cardiac arrest, and heart failure in patients with CKD (34).

Cellular senescence is a process where cells gain the maximum capacity of division and lose their division potential in somatic cells (35, 36). Some inflammatory chronic diseases are considered age-related diseases such as CKD and CVD, which are associated with kidney failure and vascular and valvular heart disease (36, 37) due to imbalance of oxidative stress, pro-inflammatory factors, and DNA damage that facilitate the accumulation of senescent cells (21, 37–39). Vascular aging is a consequence of premature aging in CKD that mediates medial VC that is a hallmark of senescence (37). Moreover, vascular and valvular heart diseases are associated with accelerated aging, accumulation of senescence, and increased inflammation that feedback these age-associated diseases and promote aortic calcification and calcific aortic valve diseases (CAVD) (36). CVD is the cause of death in 40% of the elderly (40).

Nowadays, few data describe the mechanisms/pathways by which EVs from vascular senescent cells mediate the development and progression of vascular calcification. This study compiles the role of senescent EVs from damage vasculature and the pathways and mechanisms described to date. However, more research is needed in this field. We know so far, the crossover between senescent EVs and VC has been less studied; therefore, the study of the EVs cargo generated by senescent cells and the signaling pathways in VC generation and progression could be critical in the VC prevention and treatment. In this way, senescent EV characterization and quantification could be a useful prognostic marker and therapeutic tool. This review explored the senescence-associated changes in EVs contributing to VC disease. However, due to the limited research, the work highlighted the background about this field described to date. Further studies should elucidate the role of senescent EVs in the mechanism implied in VC generation and progression.

## Development of Vascular Calcification

In the last two decades, VC was identified as a manifestation of atherosclerosis associated with diabetes, hypertension, and dyslipidemia (41–45). Various therapeutic measures have been developed to treat the associated risk factors. However, results have been unsuccessful because VC pathophysiology remains poorly understood (8). Current knowledge of VC mainly indicates that it is caused by the progression of chronic silent inflammatory diseases (without clinical symptoms at the beginning) such as atherosclerosis, CKD, and CVD, among others (46, 47) and becomes manifest when calcification is advanced and irreversible (48). When finally diagnosed, there is no treatment; therefore, arterial calcification is considered a silent disease with no clinical symptoms or signs (18, 27, 46).

The strong correlation between VC and systemic inflammation has been described (49, 50). VC develops due to disturbances in the complex and subtle balance between inhibitors and promoters, acting at both systemic and local levels (5). Calcification involves proteins and mineralization mediators similar to those that regulate the ossification process (18). Inflammatory mediators, oxidative stress, and EVs induce the dedifferentiation of vascular smooth muscle cells (VSMCs) and cause endothelial damage (51), promoting the appearance of senescent cells in the vascular wall (18, 27, 52). Therefore, patients at a high risk of developing VC may experience cardiovascular events.

Recent evidence also suggests that an imbalance in the gut microbiota generates an accumulation of bioactive metabolites in the blood and activates cellular and molecular signaling, thereby disrupting homeostasis, and promoting diseases such as CKD. Moreover, CKD progression is often accompanied by VC that is linked to the dysregulation of gut microbiota and production of harmful metabolites, such as uremic blood toxins (gut microbiota-derived metabolites in CKD patients), that promote CKD development and therefore, they are implied in the calcification process (53). As a result, endothelial damage caused by uremic toxins is associated with the progression of CKD. In addition, uremic toxins promote vascular senescence and, finally, VC. Supporting this observation, in CKD, medial arterial calcification switches the VSMC phenotype and sometimes occurs in conjunction with calcium and phosphate accumulation within atherosclerotic lesions. Thus, vascular damage leads to VC, which is triggered by uremic toxins resulting from CKD. The imbalance of the bacterial metabolism in the gut microbiota promotes the production of uremic toxins and increases the possibility of VC development (54). Therefore, the generation of uremic toxins caused by microbiota imbalance that ultimately produces vascular senescence and VC in CKD was a novel way to develop VC.

### Vascular Calcification Mediators

**FIGURE 1:**
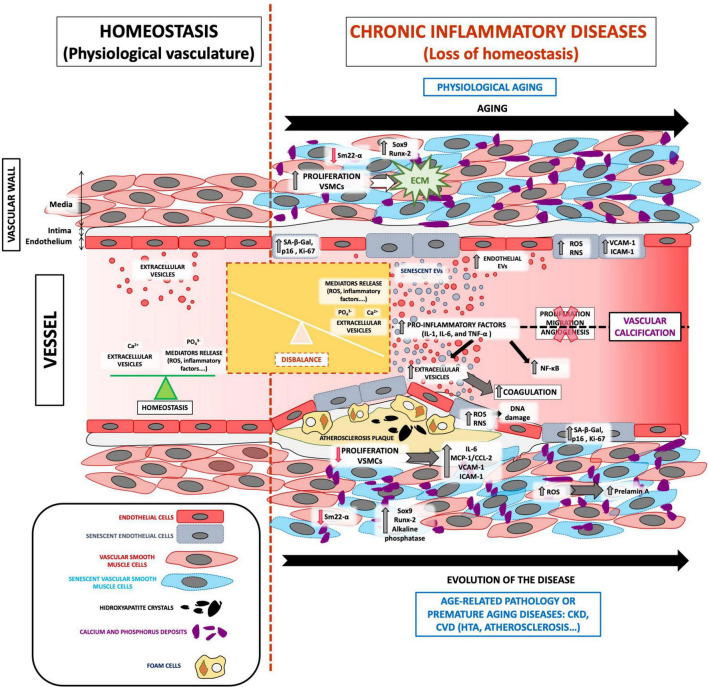
The role of EVs in VC development associated with vascular senescence. Under physiological conditions, the vasculature presents a balance between oxidative stress, inflammatory factors, and calcium and phosphate release and highlights the regular release of extracellular vesicles. All these mediators are focused on maintaining vessel homeostasis. The loss of homeostasis could appear with physiological aging and age-related pathologies such as CKD and/or CVD, which implies the imbalance of several processes. Both physiological and premature aging illnesses are characterized by an increase in ROS, calcium, and phosphate release, inflammatory mediators, adhesion molecules, coagulation process, the proliferation of extracellular matrix proliferation in the blood, and especially increased vasculature senescence. In addition, cells from a senescent vessel, especially endothelial cells and vascular smooth muscle cells, release extracellular vesicles that contain calcification factors. Complex signaling pathways in senescent extracellular vesicles cause spontaneous calcium ion precipitation in the arteries and thus contribute to VC development and pathophysiology.

The pathophysiology of VC involves multiple, complex signaling pathways that lead to mineralization (27). As already described, the loss of homeostasis leads to an accumulation of calcium and phosphate ions in the blood, causing spontaneous ion precipitation in the arteries, thereby inducing changes in the vasculature (12). VC caused by age-related pathologies, such as CKD and/or CVD, progresses in the same manner as that caused by physiological aging, but to a different extent (Figure 1).

Endothelial cells are continuously in contact with the blood in the vascular wall, and the imbalance of calcium/phosphate hampers endothelial cell function (55). Moreover, damage to endothelial cells in the vasculature generates oxidative stress through reactive oxygen species (ROS) production, which is a well-known cause of oxidative DNA damage and pro-inflammatory cytokine release (56, 57). These factors contribute actively to the appearance of extracellular matrix (ECM) deposits and increase calcium/phosphate accumulation in VSMCs, accelerating VC in CKD patients (58). It is noteworthy that damaged endothelial cells in this environment may adopt a senescent phenotype, thus contributing to VC development (18, 27).

Vascular smooth muscle cells are present alongside endothelial cells in the vasculature and are the primary cell type constituting the medial layer of the vascular wall (12). During the development of VC, VSMCs undergo dedifferentiation into osteoblast-like cells, promoting mineralization (an osteogenic transition) of the vessels. This process is associated with the upregulation of cellular and molecular targets of ROS, inflammatory factors, and EVs released from endothelial cells and VSMCs. This association highlights the multifactorial origin of VC (59, 60) occurring as a consequence of CKD (61).

### Endothelial Cell and Vascular Smooth Muscle Cell Senescence

Premature and physiological aging share many cellular phenotypes, including abnormal nuclear shape, loss of epigenetic markers, higher reactive oxygen and nitrogen species (RONS) levels, which increase lipid and protein production, cause DNA injury, increase calcium metabolism, and promote mitochondrial dysfunction (62). Cellular senescence is characterized by a stable cell cycle arrest (35) that causes inflammation and modifications of the microenvironment through the senescence-associated secretory phenotype (SASP). The SASP is a combination of molecules such as cytokines, ECM proteins, proteases, and other factors that alter the behavior of neighboring cells. Senescence is associated with phenotypic alterations that include morphological changes, such as a decrease in cellular proliferation and DNA replication. This phenotype is characterized by an increase in senescence marker levels (senescence-associated-β-galactosidase [SA-β-gal], p16^*INK*4a^, and Ki-67), lysosomal biogenesis, DNA repair protein levels, DNA injury, loss of cellular functionality, secretion of pro-inflammatory factors (IL-1, IL-6, and TNF-α), modulation of cell cycle arrest proteins, and reorganization of chromatin into discrete foci (21, 38).

In addition, senescence is exacerbated by reduced levels of renoprotective factors such as Klotho, vitamin D, and bone morphogenetic proteins (BMPs), and downregulated renoprotective mechanisms such as mitophagy (63). In this sense, senescence can be considered an adaptive cellular response to the external microenvironment. Accordingly, the interaction between endothelial cells and VSMCs in VC has been recently identified as an important factor; blocking the senescence process can attenuate osteogenic transformation (64).

Arterial aging in elderly patients leads to endothelial dysfunction, which eventually triggers a phenotypic change that results in cellular senescence (46). Endothelial cells damaged as a result of CKD may achieve a senescent phenotype characterized by a larger and flatter morphology and a polyploid nucleus, thus contributing to the evolution of VC (18, 27). Moreover, these cells exhibit changes in cytoskeleton integrity, proliferation rate, angiogenesis, and migration (21, 38). Furthermore, senescent endothelial cells demonstrate increased production of adhesion molecules (VCAM-1 and ICAM-1) and increased nuclear translocation of NF-κB. Therefore, the senescent endothelium is more susceptible to apoptosis, due to the presence of adhesion and inflammatory molecules (33, 65). Moreover, endothelial cell senescence is associated with an increase in EV release, which contributes actively to the generation and progression of VC in VSMCs (18, 21, 27). Overall, EVs from senescent endothelial cells play a critical role in VC and are considered a novel mediator.

Some reports have demonstrated that classical and novel mediators involved in VC may cause the VSMC phenotype to switch to an osteoblast-like phenotype in aortic vessels (12) and that senescent VSMCs generate a synergistic effect on the surrounding environment during the phenotypic transition. During this transition, the expression of some osteogenic/chondrogenic markers is modified. An early event in the VC signaling pathway is the downregulation of Sm22-α (a VSMC-specific marker) expression, while the expression of osteogenic/chondrogenic genes such as *Sox9* and *Runx-2* is upregulated (12, 18, 27). In this regard, VC in patients with CKD is not fully understood. It has been hypothesized that mediators such as oxidative stress, inflammation, and EVs are associated with a chronic inflammatory environment that regulates mineral metabolism in CKD patients (51, 66, 67).

Leopold et al. (68) reported that prelamin A levels increase in senescent cells through a mechanism involving ROS and pro-inflammatory cytokines, which are directly implicated in VC pathogenesis. Furthermore, it has been reported that atherosclerotic plaques increase morbidity and mortality rates in patients with advanced CKD and foster the development of atherosclerosis-related VC. This finding is associated with the development of VC in patients with CVD-associated chronic renal failure. Benz et al. (51) sought effective treatment to inhibit calcification mechanisms under CKD conditions. This finding focuses on atherosclerosis therapies to avoid calcification associated with CKD and these authors also highlighted the role of EVs in promoting inflammation and CV in CKD. Other studies that investigated VC in a model of accelerated aging reported that older subjects and younger CKD patients both presented phenotypically aged vascular walls, which was associated with upregulated prelamin A expression in calcified VSMCs (69–71). Moreover, senescent VSMCs present upregulated expression of Runx-2 and alkaline phosphatase, which are osteoblast transcription factors that enhance the transition of senescent VSMCs to the osteoblast-like phenotype and, therefore, promote calcification in vascular vessels (3, 72).

Furthermore, senescent cells in the vascular wall generate an imbalance of phosphate in VSMCs, thus promoting the pro-calcification phenotype of senescent vascular cells. Similarly, atherosclerosis is associated with premature cellular senescence; existing evidence demonstrates the presence of senescent VSMCs and atherosclerotic plaques in young patients (73). Senescent VSMCs exhibit decreased proliferation and a reduced capacity to repair plaques, with increased production of pro-inflammatory chemokines and adhesion molecule mediators (IL-6, MCP-1/CCL-2, VCAM-1, and ICAM-1) (74). Thus, inflammatory cells proliferate in this particular landscape.

## Role of Extracellular Vesicles in Vascular Calcification

Extracellular vesicles can be found in many body fluids, including plasma and urine. EVs are involved in physiological and pathophysiological processes through their involvement in the intercellular communication system. Under physiological conditions, EVs are involved in paracrine and/or endocrine communication mechanisms because they transmit biological signals to neighboring cells (75). In addition to delivering their cargo to nearby targets, EVs facilitate long-distance communication, thus regulating different biological and pathophysiological functions (76). Under pathological conditions, EVs participate in crucial processes such as inflammation, cell proliferation, and the immune response (38, 76–78). In addition, EV delivery is considered a biomarker *per se* and can be utilized to develop therapeutic strategies (76).

Indeed, EVs biogenesis, shedding and uptake, as well as their cargo content is known to be redox sensitive (79). In particular, senescence and oxidative stress promote EVs release (80–82), and recently, EVs are being included as part of the SASP (83). However, their specific content can include either antioxidants or ROS-generating enzymes; thus, oxidative-stress released EVs can trigger both antioxidant and pro-oxidant responses, thereby modulating the redox status of recipient cells (84). These EVs can also carry waste products, such as oxidized molecules, which would induce autophagy in target cells (85). Environmental factors influence the number and content of EVs in the development and progression of diseases (18, 27, 86). Levels of EVs are elevated in patients with vascular, metabolic, pulmonary, autoimmune, and neurodegenerative diseases, chronic inflammation, and cancer (87). Furthermore, an increase in EV levels is generated in vascular endothelial cells during atherosclerosis, due to stress (88). Hence, EVs are attracting increasing attention as markers for predicting, diagnosing, and monitoring complex diseases, with the potential to contribute to the identification of new therapeutic targets (88).

Some studies demonstrated that cells that arise SASP release different amounts of EVs compared with non-senescent cells and have also been shown that EVs from senescent cells present different EV cargo (18, 27, 82, 89). Moreover, these changes in EVs released by senescent cells are associated with an increase in the EV size and ultrastructural changes observed by electronic microscopy (18, 27). Furthermore, some evidence demonstrated that proteins expressed on the surface of EVs released by damaged cells is different from non-damaged cells such as cancer (90, 91) CVD (92), among others.

Extracellular vesicles general content includes calcification-promoting factors such as annexins, BMPs, and calcium. A recent study showed that senescent-cell-derived EV cargo is high in calcium, annexin A2, annexin A6, and BMP2 (18, 93). When VSMCs are cultured in the presence of EVs derived from senescent endothelial cells (93, 94), they undergo dedifferentiation (18), with a decrease in Sm22-α protein levels (27, 95). Recent evidence also suggests that EVs from the plasma of elderly subjects promote calcification in vascular muscle cells *in vitro*. Accordingly, EVs from senescent endothelial cells are involved in VC (18).

Chronic kidney disease is characterized by the accumulation of uremic toxins in the blood. Senescent endothelial cells stimulated by uremic toxins produce more EVs per cell (27). EVs produced by senescent endothelial cells, generated by treatment with primary plasma uremic toxin, indoxyl sulfate, and uremic serum from rats, cause calcification of the vasculature *in vitro* (27, 95). In the present study, senescent endothelial EV cargo was increased in calcium levels (27). The mechanism that senescent endothelial EVs generated in VSMCs mediated VC is remarkable. Recently, Alique et al. reported that senescent EVs generated an increase in Runx2 and BMP2 expression in VSMCs during VC progression (27). Moreover, these VSMCs change the phenotype to the procalcifying vascular phenotype indicated by a decrease in Sm22-α levels. During the development of this phenotype, vascular cells expressing different inflammatory cytokines such as TNF-α, TWEAK, MCP-1/CCL-2, CCL5, and IL-6 are implicated in VC (27). Furthermore, EVs play an essential role in angiogenesis, and together with their role in the development of VSMC senescence and VC generation, they mediate CKD progression and associated cardiovascular complications (96). Moreover, EVs in CKD patients have been proposed as therapeutic targets (97). Thus, the effect on the initiation and progression of VC depends on the number and cargo of EVs generated in patients with chronic inflammatory pathologies.

Recent evidence showed that EVs from senescent endothelial cells are implied in the VC (18, 27). Furthermore, some microRNAs are mediated in the endothelial senescence, highlighting the downregulation of miR126-3p, miR126-5p, miR21-3p, miR155, and miR210, critical keeping endothelial homeostasis. Therefore, finally, cellular damage carries endothelial cells to achieve the senescent phenotype (27). Moreover, as a consequence, senescent endothelial EVs release an increase of calcium and magnesium compared with “young” endothelial EVs (27). Moreover, it has been demonstrated that senescent cells suffer DNA damage that increases EV release in cancer (98). Finally, Wallis et al. (83) showed changes in EV cargo in senescent cells depending on their content, and senescent EVs have a different role, harmful or beneficial.

Vascular smooth muscle cells undergoing calcification may release calcifying EVs themselves (22), which contain metalloproteinase-2 (MMP-2); annexin A2, A5, and A6; and phosphatidylserine (PS) on the surface. The enrichment of these matrix EVs enhances calcium-binding, coagulation, hydroxyapatite formation, and subsequently, calcification of the vascular walls in aging-related calcification (55). Accumulating evidence suggests that senescent endothelial EVs act as promoters of VC, initiating a cascade of events that cause vascular injury and finally, CVD development (38, 99). The production of EVs by senescent endothelial cells is considered a pathogenic mechanism of endothelial dysfunction (21). Endothelial EVs promote damage to VSMCs and the vascular endothelium (38, 39). In this context, both CVD in the elderly and chronic diseases in younger patients cause vascular senescence and VC (1, 63).

Notably, the relationship between EVs, VC, and senescence is unclear. To date, a search in PubMed using the keywords “extracellular vesicles,” “vascular calcification,” and “senescence” gave 24 results (Table 1). Interestingly, 15 of the 24 results are reviews and only 8 are original research articles. The first study was published in 2014. More research is needed to continue unraveling the complex underlying mechanism that correlates all three factors.

**TABLE 1:** Original research articles and reviews obtained after a search in PubMed with the following keywords: “extracellular vesicles” and “vascular calcification” and “senescence.”

Type of article	Publication year	References	Model	Experiment (tissue/cells)	Findings/Results
Research	2021	(100)	*In vivo*	EVs pooled from the human whole tissue proteome and miRNAome (carotid artery plaque and calcified aortic valve)	71 proteins and 5 miRNAs were significantly altered between the artery and valve EVs
Research	2021	(101)	*In vivo* *In vitro*	The thoracic aorta of WT rat aortas Tissue from human carotid arteries and human aortic Human aortic VSMCs	Warfarin increased vascular calcification in an endoplasmic reticulum stress-dependent manner *via* increased EVs release
Research	2021	(102)	*In vitro* *In vitro*	Human aortic vascular smooth muscle cells EVs from bone mesenchymal stem cell	EVs from bone mesenchymal stem cell Inhibition of VSMCs calcification
Research	2020	(103)	*In vitro* *In vivo*	Human VSMCs 5/6-nephrectomy + high phosphate diet mice	EVs from melatonin-treated VSMCs attenuate VC and aging in VSMCs and mice
Research	2020	(27)	*In vitro* *In vitro*	Human endothelial cells Human VSMCs	EV from indoxyl sulfate-treated endothelial cells generate calcification in VSMCs
Research	2019	(104)	*In vitro* *In vitro*	Human endothelial cells Human VSMCs	EVs from high glucose-treated endothelial cells induce calcification in VSMCs
Research	2017	(18)	*In vivo* *In vitro* *in vitro*	EVs from elderly EVs from senescent human endothelial cells Human VSMCs	EVs of senescent endothelial cells and EVs from plasma of elderly subjects promote vascular calcification (in VSMCs)
Research	2015	(17)	*In vivo* *In vitro* *in vitro*	EVs from plasma of CKD patients EVs from TNF-α-treated human endothelial cells Human VSMCs	EV from TNF-α-treated endothelial cells and EV from plasma of CKD subjects promote vascular calcification (in VSMCs)

**Type of article**	**Publication year**	**References**	**Title**

Review	2022	(105)	Matrix vesicles as a therapeutic target for vascular calcification		
Review	2021	(106)	Exosomes and melatonin: Where their destinies intersect		
Review	2021	(100)	Calcifying extracellular vesicles as building blocks of microcalcifications in cardiovascular disorders		
Review	2020	(107)	Omics research in vascular calcification		
Review	2019	(108)	Cardiovascular calcification: artificial intelligence and big data accelerate mechanistic discovery		
Review	2019	(22)	The interplay between mineral metabolism, vascular calcification and inflammation in chronic kidney disease (CKD): Challenging old concepts with new facts		
Review	2019	(1)	Multifaceted mechanisms of vascular calcification in aging		
Review	2019	(109)	[Molecular mechanism of vascular calcification] [Article in Japanese]		
Editorial	2019	(110)	A dual role for GRP in cardiovascular disease		
Review	2018	(4)	Role of smooth muscle cells in vascular calcification: implications in atherosclerosis and arterial stiffness		
Review	2018	(111)	Exosomes, the message transporters in vascular calcification		
Review	2018	(21)	Senescent microvesicles: A novel advance in molecular mechanisms of atherosclerotic calcification		
Review	2017	(112)	Vascular calcification in CKD-MBD: Roles for phosphate, FGF23, and Klotho		
Review	2016	(113)	Vascular calcification in uremia: New-Age concepts about an old-age problem: Methods		
Review	2015	(114)	[Vascular Calcification–Pathological Mechanism and Clinical Application–Mechanisms of vascular calcification]: [Article in Japanese]		

## Conclusion

The incidence of VC is increasing in developed countries, and VC can significantly increase cardiovascular risk. The processes and mechanisms involved in VC are unclear, and new therapeutic strategies are needed to prevent or reverse calcification. EVs have been outlined as a mediator in VC development, especially those released by senescent vasculature cells. Moreover, EVs are mediators that regulate aortic valve calcification evolution. Patients with CKD have a high prevalence of vascular calcification. To develop early diagnostic methods, evaluating EVs’ role in aging and age-related diseases such as CKD and VC is necessary. Consequently, studying EVs from damaged vasculature in physiological aging and age-related diseases to prevent the progression of VC is essential. It highlights EVs’ implication in the development of VC to develop early diagnostic methods to treat elderly and premature aging diseases (such as CVD associated with CKD) that will be achieved through VC.

## Author Contributions

MA: conceptualization, methodology, investigation, resources, and writing–original draft preparation. CM-B, CB, and MA: review and editing. CB and MA: funding acquisition. All authors have read and agreed to the published version of the manuscript.

## Conflict of Interest

The authors declare that the research was conducted in the absence of any commercial or financial relationships that could be construed as a potential conflict of interest.

## Publisher’s Note

All claims expressed in this article are solely those of the authors and do not necessarily represent those of their affiliated organizations, or those of the publisher, the editors and the reviewers. Any product that may be evaluated in this article, or claim that may be made by its manufacturer, is not guaranteed or endorsed by the publisher.
